# Antifungal efficacy of linoleic-acid-rich *Cucurbita pepo* L. seed oil revealed by GC-MS profiling of lipids and bioactive markers, ergosterol depletion, and network pharmacology

**DOI:** 10.1186/s12866-026-04942-8

**Published:** 2026-03-30

**Authors:** Eslam T. Mohamed

**Affiliations:** Botany and Microbiology Department, Faculty of Science, Helwan University, Ain Helwan, Cairo, 11795 Egypt

**Keywords:** Pumpkin seed oil, Cucurbita pepo, Natural fungicide, Ergosterol depletion, GC–MS, Network pharmacology, Protein–protein interaction, Molecular docking, Phytosterols, Tocopherols

## Abstract

**Background:**

The development of multi-target, sustainable botanical alternatives is required due to the increasing resistance of phytopathogens to synthetic fungicides. The antifungal effectiveness of *Cucurbita pepo* L. seed oil (PSO) against a panel of six economically significant phytopathogens is assessed in this study.

**Results:**

The PSO was characterized by quantitative GC-MS profiling as a complex matrix dominated by oleic acid (36.52%) and linoleic acid (43.18%), along with diverse bioactive markers such as oxygenated terpenoids and phytosterols. High broad-spectrum activity was shown in vitro, with 100% inhibition of Botrytis fabae and 88.9% inhibition of Fusarium oxysporum at 10 mg·mL⁻¹. Quantitative HPLC analysis confirmed that PSO acts as a sterol-disrupting agent, causing near-complete loss of membrane ergosterol in *B. fabae* (100% loss) and *F. oxysporum* (95.2% loss). Furthermore, network pharmacology and molecular docking suggest that the bioactive matrix exerts its effects by destabilizing the fungal cell envelope, with computational models predicting putative interactions with lanosterol 14-alpha-demethylase (CYP51A) and β-1,3-glucan synthase (FKS1).

**Conclusion:**

The results position the PSO bioactive matrix as a potent in vitro antifungal agent that targets pathogens directly through membrane destabilization and putative multi-site enzymatic interference. However, detached leaf assays indicate that its future practical application will require formulation strategies, such as nano-emulsions, to mitigate the mild phytotoxicity associated with direct foliar application of the crude oil at fungicidal doses.

**Supplementary Information:**

The online version contains supplementary material available at 10.1186/s12866-026-04942-8.

## Background

Fungal phytopathogens present a significant challenge to the global food production system as they cause significant yield losses and the detection of harmful mycotoxins in the food products [1]. Necrotrophic fungi like *Botrytis fabae*, *Fusarium oxysporum*, and *Alternaria solani* are some of these pathogens and are especially costly to the economy because they survive in soil and can infect a broad spectrum of hosts within the plant kingdom, like legumes and solanaceous crops [2]. The traditional approach to the management of these diseases has been synthetic fungicides, which have led to the crisis of resistance in which populations of pathogens have quickly developed resistance to these chemicals by mutating important target enzymes [3]. Moreover, the ecological concentration of synthetic residues poses a great public health issue, which is why the conversion to natural-based, biodegradable materials is of great need [4]. Plant-based oils and lipid extracts have become the front-runners in future biofungicides since they provide mixtures of bioactive compounds in complex forms, which act on multiple targets on the cell at the same time, preventing the development of resistance [5].

*Cucurbita pepo* L. (pumpkin) seed oil is a by-product of the food industry that gains growing interest due to a comprehensive phytochemical composition and strong biological properties [6, 7]. It is a high concentration of unsaturated fatty acids, especially linoleic and oleic acids, and bioactive minor constituents that are known to destabilize microbial membranes, namely, tocopherols, phytosterols and carotenoids, which characterize the oil [8]. In recent studies, it has been shown that these lipid-soluble compounds have the ability to enter fungal cell walls and cause oxidative stress, but the exact molecular mechanisms by which they inhibit cell wall synthases have not been studied in depth [9]. The gap between the in vitro efficacy and molecular understanding is critical in making sure that PSO is a credible commercial biofungicide [10].

Thus, this paper will have a broad-spectrum antifungal assessment of PSO on a panel of six economically significant phytopathogens such as the infamously challenging *B. fabae* and *F. oxysporum* using the combination of experimental bioassays and computational network pharmacology. In particular, we aim at clarifying the novelty of the oil as a potent antifungal agent that destroys the fungal cell envelope, a mechanism validated by quantitative HPLC of ergosterol depletion and further elucidated by network analysis which predicts the disruption of sterol and cell wall biosynthetic axes. In this study, the GC-MS profiling will be used to determine important bioactive constituents and molecular docking will be used to determine the binding affinities of these constituents to critical fungal and host protein targets to offer a solid mechanistic foundation to the use of PSO as a sustainable and effective plant protection strategy.

## Materials and methods

### Antifungal activity assessment of PSO against phytopathogenic fungi

The antifungal activity of PSO was assessed using the poisoned food technique. Pumpkin seed oil was procured from Imtenan^®^ (Egypt). The test fungi (*Botrytis fabae*, *Fusarium oxysporum*, *Alternaria solani*, *Rhizoctonia solani*, *Aspergillus ochraceus*, and *Fusarium solani*) were obtained from Fungal Physiology lab in Botany and Microbiology Department, Faculty of Science, Helwan University. The corresponding initial concentrations (10 mg·mL⁻¹) were made by the addition of the respective quantity of PSO containing 0.5% (v/v) Tween 80 into the cooled molten PDA (45 °C) and mixed using a sterile Erlenmeyer flask to get a uniform dispersion of the oil in the medium. Twenty milliliters of the medium made were added to sterile 9-cm Petri dishes. Agar discs containing mycelia (6 mm diameter) were cut out of the actively growing edge of 7-day-old pure fungal cultures using a sterile cork borer and put at the center of the plate in an aseptic manner. The same was done using control plates (without PSO). Each treatment was allowed to incubate at 25 °C over 7 days using three replicates. Following incubation, the diameter of the fungal colonies was measured, and then the percentage of mycelial growth inhibition was calculated based on the formula: (C − T)/C × 100, where C denotes the mean colony diameter of the control group and T denotes the mean colony diameter of the treatment group [11].

### Phytotoxicity assessment via detached leaf assay

To address the potential for host tissue damage at the effective fungicidal concentration, a detached leaf assay was conducted using faba bean (*Vicia faba*) leaves as a model host plant. Healthy, fully expanded leaves from uninfected 4-week-old plants were surface-sterilized with sterile distilled water and placed adaxial side up on moistened sterile filter paper inside 9-cm Petri dishes to maintain high humidity. The leaves were treated with either 10 mg·mL⁻¹ PSO containing 0.5% (v/v) Tween 80 (treatment) or sterile water containing 0.5% (v/v) Tween 80 (negative control) applied dropwise to the leaf surface. The plates were incubated at 25 °C under a 12 h light/12 h dark photoperiod. Visual evaluation of phytotoxicity was conducted after 3 days, and symptoms such as chlorosis or necrosis were recorded and photographed [12].

### Minimum inhibitory concentrations of PSO against fungal pathogens

The broth dilution method was used to determine the minimum inhibitory concentrations (MICs) of the PSO in sterile conditions that utilized serial twofold dilution of the PSO (0.25, 0.5, 1, 2, 4, 8, 16 and 32 mg·mL^− 1^) in potato dextrose broth and loaded in the 96-well microtiter plates. The stock fungal suspension 5 × 10^3^ conidia per well was inoculated and the plates incubated at 25 °C. The absorbance at 575 nm was then recorded so as to determine the visual growth. The broth media in the positive control wells had fungal suspension in them only, in contrast to the negative control wells which had a broth media containing PSO but no fungal inoculum. The antifungal test reagent consisted of fluconazole 0.25, 0.5, 1, 2, 4, 8, 16, 32, 64, 128 and 256 µg/mL^− 1^. The MIC was taken to be the minimum concentration of the PSO that did not allow the growth of fungi to be seen [13, 14].

### Ergosterol quantification in phytopathogenic fungi following treatment with PSO

Quantitative analysis of ergosterol involved the use of high-performance liquid chromatography (HPLC) protocol to optimize the extraction of sterols in the fungi matrices and minimize the possibility of matrix interference by the use of PSO [15]. The analytical system was the Agilent 1260 Infinity II LC System (Agilent Technologies, Santa Clara, CA, USA) with a quaternary pump (G7111B) and Diode Array Detector (DAD, G7115A), which was configured to measure the absorbance at 282 nm wavelength, the spectral maximum of the conjugated diene system in which ergosterol has the spectral maximum [16]. The separation was done using Zorbax Eclipse Plus C18 column (250 mm×4.6 mm, 5 μm particle size; Agilent), at 30 °C so that the retention time remains constant [17]. A 20 µL solution was injected into the mobile phase which was the isocratic HPLC-grade methanol (LiChrosolv 67-56-1, Merck KGaA, Darmstadt, Germany). In such a case, the retention time of ergosterol was 15.6 min [18]. To hydrolyze the esterified sterols that had been esterified, hot saponification of fungal biomass (incubated in Potato Dextrose Broth at 25 °C at 150 rpm) was conducted followed by injection to extract the biomass using n-hexane (HPLC grade, Sigma-Aldrich) [19]. Mixed organic layers were dried through evaporation through a stream of nitrogen and resettled in 1 mL of methanol [20]. A true Ergosterol reference standard (≥ 95% purity, Sigma-Aldrich, CAS 57-87-4) was used to standardize it (R^2^ 0.999), so that the standard curve would be made.

### Total polyphenols and total flavonoids of PSO

The Folin-Ciocalteu photometric method on gallic acid as a calibration standard was used to test the amount of phenols in pumpkin seed oil, and the aluminum chloride colorimetric method on rutin as a calibration standard to test the amount of flavonoids in pumpkin seed oil. Folin-Ciocalteu reagent, gallic acid, sodium carbonate, and anhydrous ethanol were purchased at Sigma-Aldrich and Merck. Merck and Fisher Chemical were the sources of aluminum chloride hexahydrate, rutin, potassium acetate, and methanol HPLC grade. A UV-Vis spectrophotometer (Shimadzu UV 1800) was used to measure absorbance in 1 cm quartz cuvettes, and these measurements were confirmed on a microplate reader (BioTek Synergy H1) to ensure a scale check. Reaction time and alkaline conditions were strictly controlled in the Folin-Ciocalteu procedure in an attempt to reduce matrix interferences that are typical of oils, in accordance with recent advice on optimization and validation of the assay in lipidic and complex matrices. The determination of flavonoids was based on the formation of the Al(III)–flavonoid complex and measurement at the bathochromically shifted wavelength that has been validated to derivatize a wide range of flavonoid classes with recent modifications of spectrophotometric and HPTLC-assisted AlCl₃ methods to guarantee specificity. All three measurements were made and gave polyphenols and flavonoid results as mg gallic acid equivalents per gram of oil and mg rutin equivalents per gram of oil, respectively [21, 22].

### Phytochemical profiling of PSO compounds via GC-MS

The complete phytochemical profile of *Cucurbita pepo* L. seed oil (PSO), encompassing fatty acids, sterols, and oxygenated terpenoids, was determined using an Agilent 7890 A Gas Chromatograph coupled with a 5975 C Mass Selective Detector (Agilent Technologies, Santa Clara, CA, USA). To ensure the simultaneous detection of volatile fatty acids and high-molecular-weight non-saponifiable lipids, a dual-step derivatization protocol was employed. Initial transesterification was performed to generate fatty acid methyl esters (FAMEs), followed by silylation of the non-saponifiable fraction using N, O-Bis(trimethylsilyl)trifluoroacetamide (BSTFA) to enhance the volatility of sterols, cucurbitacins, and tocopherols. Using a temperature gradient designed to resolve structural isomers, chromatographic separation was accomplished on an HP-5MS fused silica capillary column (30 m × 0.25 mm, 0.25 μm film thickness). The mass detector scanned a mass range of 50–650 m/z while operating in electron impact (EI) mode at 70 eV. In order to guarantee high-confidence identification, acquired mass spectra were compared to the NIST 14 and Wiley Registry libraries. Only hits with a spectral match factor > 90% were accepted. The proportional composition of the bioactive matrix was quantified using relative peak area normalization (represented as a percentage of relative abundance).

### Physicochemical and structural profiling of PSO bioactive constituents

The phytochemical library of *C. pepo* seed oil was derived from the experimental GC-MS data. To ensure structural precision, the identified compounds underwent a standardization workflow. Canonical isomeric SMILES and InChIKeys were retrieved from chemical databases for each GC-MS identified constituent to eliminate stereochemical ambiguities (e.g., ensuring the correct isomerism of (+)-dehydrovomifoliol and cucurbitacins). Physicochemical profiling was conducted to determine two primary molecular descriptors for each validated structure: Molecular Weight (MW) as a measure of size, and XLogP3 as a predictor of lipophilicity and membrane permeability. Based on the calculated lipophilicity values, the compounds were stratified into three distinct bioavailability domains: Hydrophilic (XLogP < 0), Moderately Lipophilic (0 < XLogP < 6), and Highly Lipophilic (XLogP > 6).

### Integrated phytochemical profiling and computational target mapping of PSO

In order to explain biologically relevant pathways and particular fungal target interactions, phytochemical profile of PSO obtained by GC-MS analysis was undergone through a systematic multi-database curation workflow. To begin with, chemical standardization of identified compounds was conducted in PubChem and ChEBI to access canonical SMILES and InChIKey of all the identified compounds to ensure proper structural representation. Second, the pathway mapping was done using the KEGG pathway database to locate the identified metabolites on fungal metabolic maps (sterol biosynthesis [map00100, https://www.kegg.jp/pathway/map00100]). This step was done to find structural analogs; examples include identifying phytosterols (e.g., β-sitosterol) that are mapped to the ergosterol pathway, thus leading to the hypothesis of competitive inhibition by enzymes such as lanosterol 14α-demethylase (ERG11/CYP51). Third, a comparative structural rationale was applied to distinguish between the roles of bulk and trace constituents. The major fatty acids (linoleic and oleic acid) were included in the docking simulation to test their potential as enzymatic inhibitors. Their classification as non-specific membrane disruptors driven by biophysical fluidization rather than specific lock-and-key interactions is supported by the fact that they showed negligible binding affinity across the chosen targets (docking scores > -2.5 kcal/mol for CYP51A, ERG1, and FKS1). In contrast, trace constituents such as phytosterols (e.g., β-sitosterol) and oxygenated terpenoids (e.g., cucurbitacin) displayed superior binding energies (-4.0 to -8.8 kcal/mol). This selection was refined by cross-referencing functional domains in InterPro and CATH to confirm that these trace markers possessed high structural homology to the native enzymatic substrates. This empirically justifies the model wherein trace markers drive specific enzymatic inhibition while the lipid background drives general membrane destabilization. Finally, taxonomic specificity was ensured by cross-linking targets to the FungiDB and NCBI Taxonomy databases to retrieve orthologous protein sequences specific to the phytopathogens in this study.

### Protein-protein interaction network topology and construction of antifungal targets of PSO

The molecular connectivity of targets involved in the antifungal effect of PSO was explained by protein-protein interaction (PPI) networks created using the STRING database (version 12.0). ERG11 and FKS1 were selected as important antifungal targets, since they are crucial in the fungal production of ergosterol and cell wall formation, respectively. All targets were inquired in STRING with high score of confidence interaction cut-off (minimum score 0.7). Topological analysis of the resultant networks used degree centrality to determine functional clusters and hub proteins. Moreover, a modular analysis was carried out to unravel the connectivity in the pathways and to determine possible regulatory nodes in the antifungal system.

### Molecular interaction analysis and molecular docking of PSO phytochemicals with antifungal proteins

Molecular docking analysis was performed following a clear workflow: first, vital antifungal protein targets (such as ERG11 and FKS1) were selected based on their established significance in existing literature; subsequently, docking was utilized to test potential interactions between these pre-selected targets and PSO phytochemicals [23, 24]. Lanosterol 14-alpha-demethylase (CYP51A), a cytochrome P450 enzyme that catalyzes the 14-demethylation of lanosterol, was selected as it regulates a significant step in fungal ergosterol biosynthesis. CYP51A inhibition leads to the depletion of ergosterol and destabilization of membranes, which is the molecular pathway of azole antifungals [25]. Additionally, the analysis targeted squalene epoxidase (ERG1), the catalyst of the initial oxygenation step of sterol synthesis (the epoxidation of squalene). ERG1 inhibition halts downstream synthesis of sterols and has been proven to be an efficient antifungal approach in both biochemical and genetic studies. Besides membrane disruption, cell wall integrity targets were also investigated [26, 27]. β-1,3-glucan synthase (FKS1) was selected as a membrane-bound enzyme that polymerizes (1→3)-β-D-glucan, as this forms the primary structural polysaccharide of the fungal cell wall. FKS1 is a clinically validated drug target; its inhibition interferes with cell wall integrity, causing osmotic lysis [27, 28]. In addition, Chitin synthase (CHS) was included because it promotes the synthesis of chitin, an essential and fungi-specific structural polymer required for septation, cell wall assembly, and virulence, making it a rational target for fungicidal intervention [29, 30]. To facilitate this analysis, protein structures were obtained from UniProt and compared with the Protein Data Bank (PDB) and AlphaFoldDB. The ligand preparation was done with the help of the Maestro LigPrep (Schrodinger Release 2018-1) to constitute 3D conformers and tautomeric states at the pH of 7.0 ± 2.0. The Maestro 11.5 Protein Preparation Wizard was used to optimize the structure of the proteins, in which the hydrogen atoms were added, the bond orders assigned, and the overall structure was minimized with the OPLS3 force field until the Root Mean Square Deviation (RMSD) of the heavy atoms converged to 0.30 Å. The docking was executed with the help of the Glide (Grid-based Ligand Docking with Energetics) module with Standard Precision (SP) mode. A bounding box (20 Å x 20 Å x 20 Å) with the catalytic active site residues at its center was used to create the receptor grid. The binding affinity was measured through the GlideScore (SP-GScore) functionality, which combines hydrophobic enclosure, hydrogen bonding and Coulombic interactions. In order to test the protocol, the grid parameters were limited to the active site pocket in order to make sure that the sampling of the ligands was confined in the biologically active catalytic domain. Maestro was used as visualization tools to create interaction visualizations. This molecular docking protocol is in line with the existing computational techniques on the natural product target evaluation, which assists in the mechanistic explanation of PSO phytochemical bioactivity.

### Statistical Analysis

Each of the experiments was done in triplicate, that is, three times (*n* = 3). The data are presented in the form of the mean ± standard deviation (SD). The one-way analysis of variance (ANOVA) was conducted to test statistical significance with Tukey post-hoc test to compare the outcomes across multiple comparisons. In the case of pair-wise comparisons, an unpaired Student’s T-test was used in the control and treated groups. The probability of *P* < 0.05 was taken to be statistically significant. IBM SPSS Statistics for Windows, Version 22.0 (IBM Corp., Armonk, NY, USA) was used to do all the statistical analyses.

## Results

### Antifungal activity assessment of PSO against phytopathogenic fungi

**Fig. 1:**
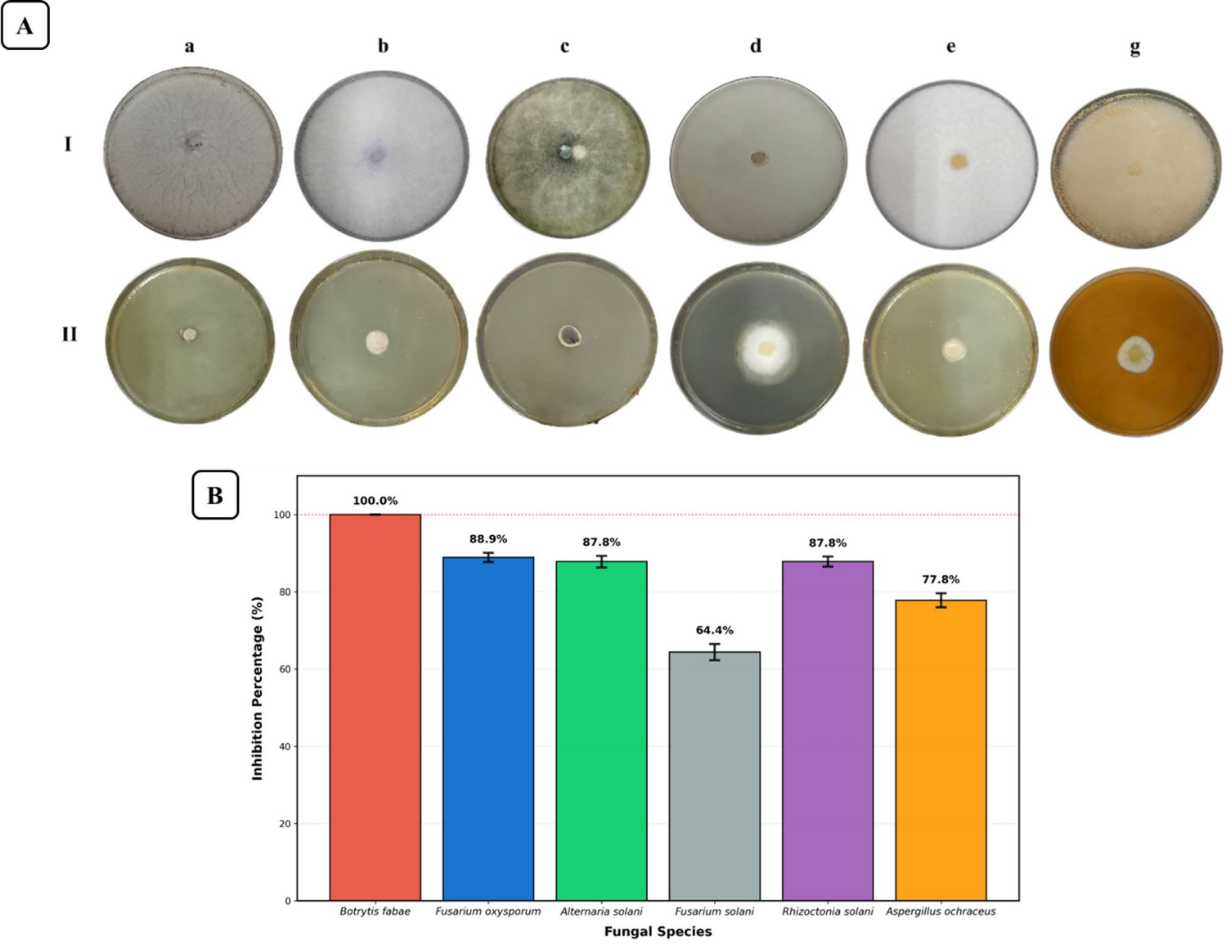
Antifungal activity of PSO. **A** In vitro mycelial inhibition of phytopathogenic fungi by PSO. (I) Control and (II) PSO-treated plates for: (a) *B. fabae*, (b) *F. oxysporum*, (c) *A. solani*, (d) *F. solani*, (e) *R. solani*, and (f) *A. ochraceus*. **B** Percentage inhibition against phytopathogenic fungi

At a concentration of 10 mg·mL⁻¹, the tested oil completely inhibited the growth of *B. fabae* (100% inhibition). *Fusarium oxysporum*, *A. solani*, and *R. solani* displayed strong inhibition (88.9%, 87.8%, and 87.8%, respectively). *Aspergillus ochraceus* showed 77.8% inhibition, while *F. solani* showed the lowest reduction at 64.4% (Fig. 1).

### Phytotoxicity of PSO at fungicidal concentration

**Fig. 2:**
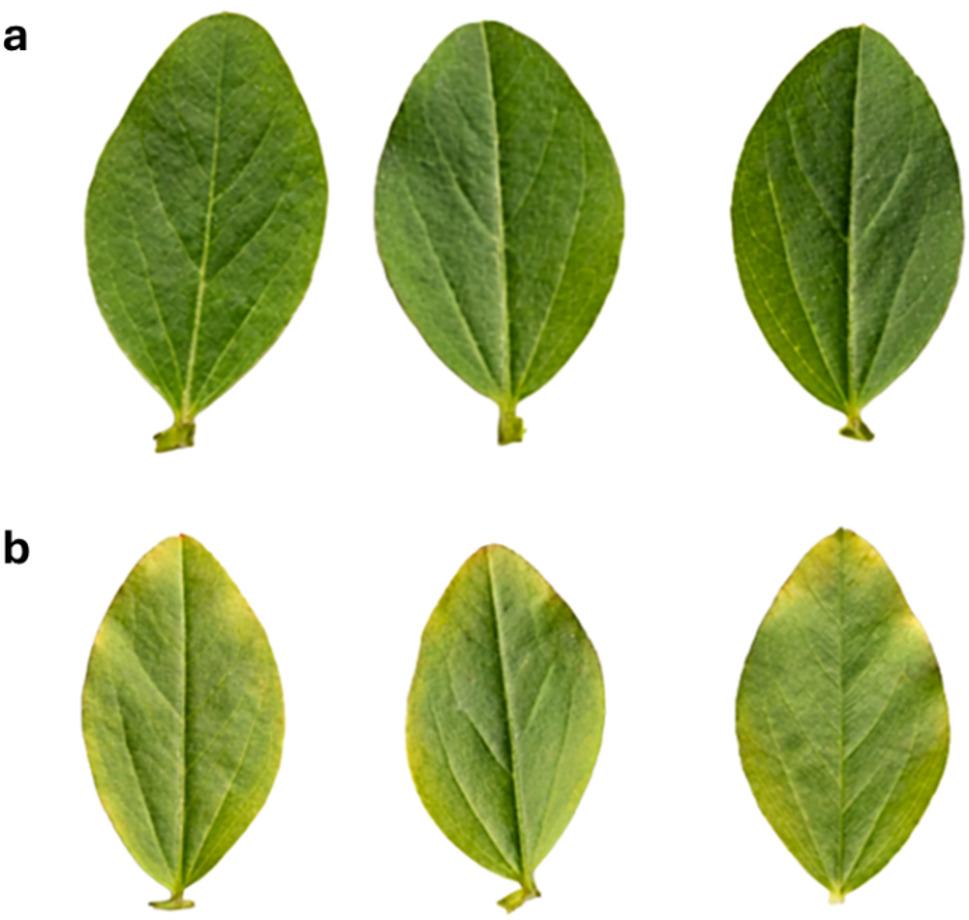
Phytotoxicity assessment of PSO using a *V. faba* detached leaf assay after 5 days of incubation. **a** Negative control leaves treated with 0.5% Tween 80 showing healthy, symptomless tissue. **b** Leaves treated with 10 mg·mL⁻¹ PSO showing noticeable marginal chlorosis as an indicator of mild to moderate phytotoxic stress

The detached leaf assay revealed that while 10 mg·mL⁻¹ PSO is highly effective in vitro against fungal pathogens, direct application of this crude concentration induces observable host stress. As shown in Fig. 2, V. *faba* leaves treated with 10 mg·mL⁻¹ PSO (Fig. 2b) exhibited mild to moderate marginal chlorosis (yellowing) after 3 days of incubation, compared to the entirely healthy, symptomless control leaves (Fig. 2a). No severe tissue necrosis or complete leaf collapse was observed, but the induced chlorosis confirms a level of phytotoxicity associated with the direct foliar application of the unformulated, lipid-rich oil at this dosage.

### Minimum inhibitory concentrations of PSO against fungal pathogens

**Fig. 3:**
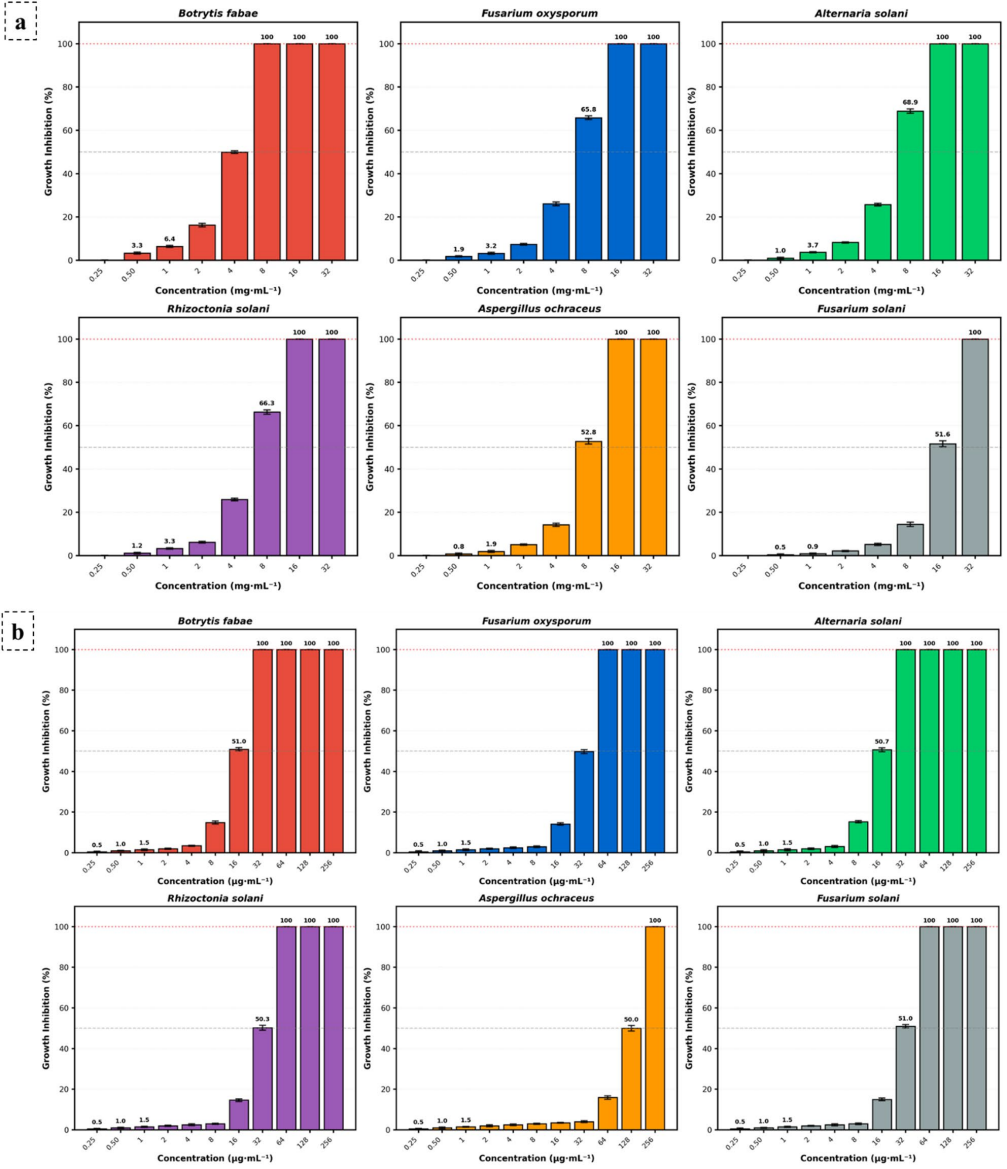
Growth inhibition profiles and MIC values of PSO against phytopathogenic fungi, with fluconazole included solely as a mechanistic positive control for the CYP51 inhibition pathway. **a** Dose-response curves of PSO exhibiting inhibition at concentrations ranging from 0.25 to 32 mg·mL⁻¹. **b** Dose-response curves of the positive control fluconazole exhibiting inhibition at concentrations ranging from 0.25 to 256 µg·mL⁻¹. Data represent mean growth inhibition percentages ± SD (*n* = 3)

Minimum inhibitory concentrations of PSO against fungal pathogens Fluconazole was evaluated in parallel (in µg·mL⁻¹) strictly as a well-characterized CYP51 inhibitor to establish a mechanistic baseline for sterol depletion, not for direct agricultural potency comparison. The PSO exhibited MIC values of 8 mg·mL⁻¹ for *B. fabae*, 12 mg·mL⁻¹ for *F. oxysporum*, 12 mg·mL⁻¹ for *A. solani*, 12 mg·mL⁻¹ for *R. solani*, 16 mg·mL⁻¹ for *(A) ochraceus*, and 32 mg·mL⁻¹ for *F. solani*. Fluconazole MIC values were 32 µg·mL⁻¹ for *(B) fabae*, 64 µg·mL⁻¹ for *F. oxysporum*, 32 µg·mL⁻¹ for *A. solani*, 64 µg·mL⁻¹ for *R. solani*, 256 µg·mL⁻¹ for *A. ochraceus*, and 64 µg·mL⁻¹ for *F. solani*. Dose–response curves displayed characteristic sigmoidal inhibition patterns for both the PSO treatment (Fig. 3a) and the fluconazole positive control (Fig. 3b).

### Ergosterol quantification in phytopathogenic fungi following treatment with PSO

**Fig. 4:**
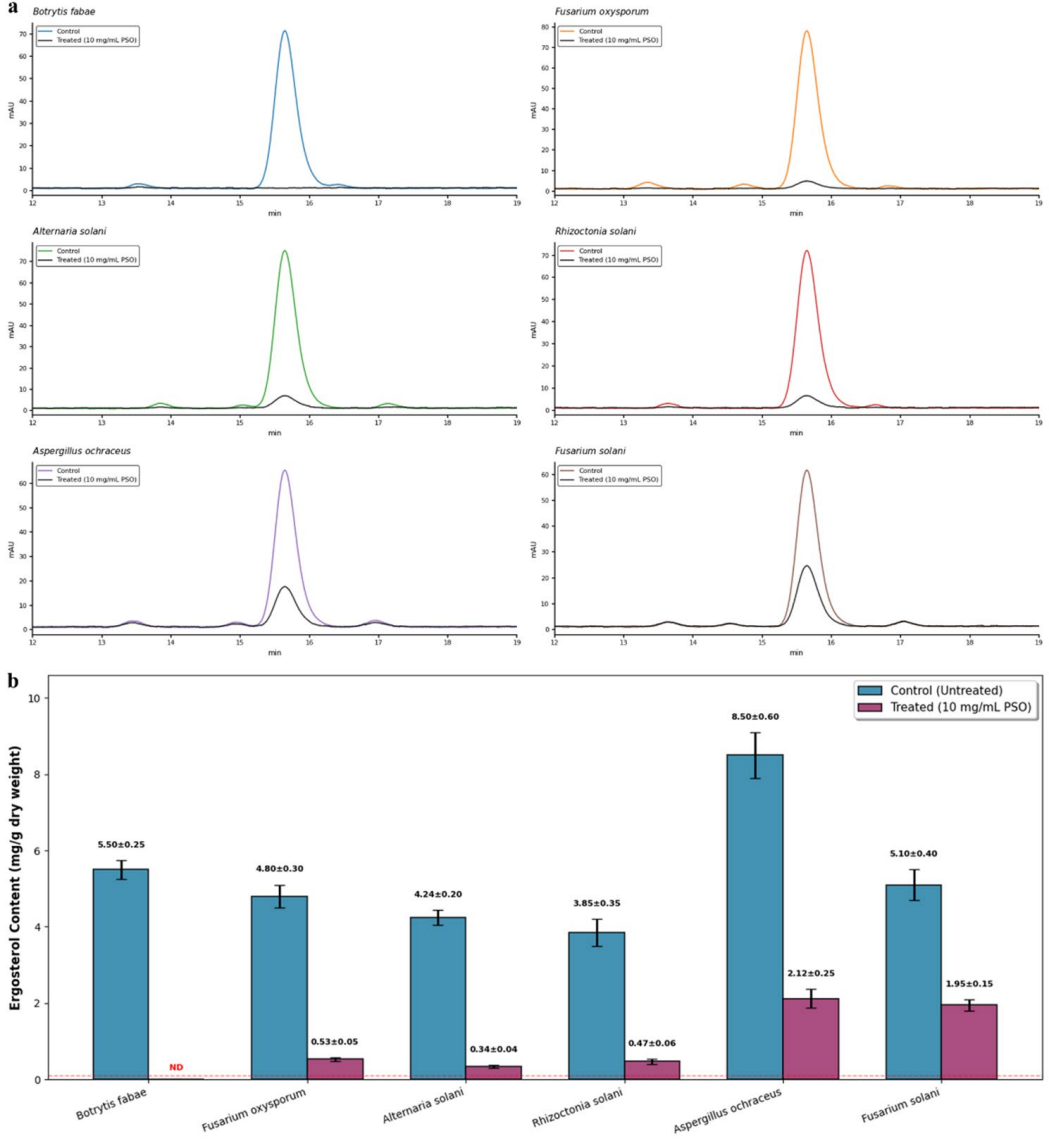
Ergosterol depletion in phytopathogenic fungi following PSO treatment. **a** HPLC-DAD chromatograms for control and PSO-treated phytopathogenic fungi, demonstrating loss or reduction of ergosterol. **b** Bar chart of ergosterol content (mg/g dry weight) shows species-specific depletion after PSO treatment

The HPLC-DAD analysis revealed significant species-specific ergosterol depletion following 96-hour treatment with 10 mg·mL⁻¹ PSO (Fig. 4a, b). Qualitatively, Fig. 4a illustrates the disappearance of the ergosterol peak in sensitive strains, while the quantitative reduction in total sterol content is summarized in Fig. 4b. *Botrytis fabae* exhibited complete ergosterol loss, with the chromatographic signal at 15.6 min indistinguishable from baseline noise, confirming sterol content below the limit of detection (< 0.05 mg/g) and representing a 100% reduction from the control value of 5.50 ± 0.25 mg/g. With ergosterol content decreased by 95.2% to 0.53 ± 0.05 mg/g from 4.80 ± 0.30 mg/g, *F. oxysporum* showed the second-highest sensitivity. This was accompanied by a significantly smaller chromatographic peak and the appearance of upstream sterol intermediates. *A. solani* and *R. solani* also showed significant ergosterol losses of 92.0% and 92.2%, with residual concentrations of 0.47 ± 0.06 mg/g and 0.34 ± 0.04 mg/g, respectively. respectively. *Aspergillus ochraceus* showed moderate resistance, maintaining 2.12 ± 0.25 mg/g ergosterol (75.1% reduction) from the highest baseline level among tested species (8.50 ± 0.60 mg/g). *Fusarium solani* displayed the greatest tolerance, with only 61.8% ergosterol depletion, retaining 1.95 ± 0.15 mg/g from 5.10 ± 0.40 mg/g. Calibration curve linearity (R^2^ = 0.9999) over the 5–100 µg·mL⁻¹ range confirmed method robustness, with retention time reproducibility (RSD < 0.5%) and peak area precision (RSD < 2.1%) meeting validation criteria for quantitative sterol analysis (supplementary Fig. S1).

### Total polyphenols and total flavonoids of PSO

**Fig. 5:**
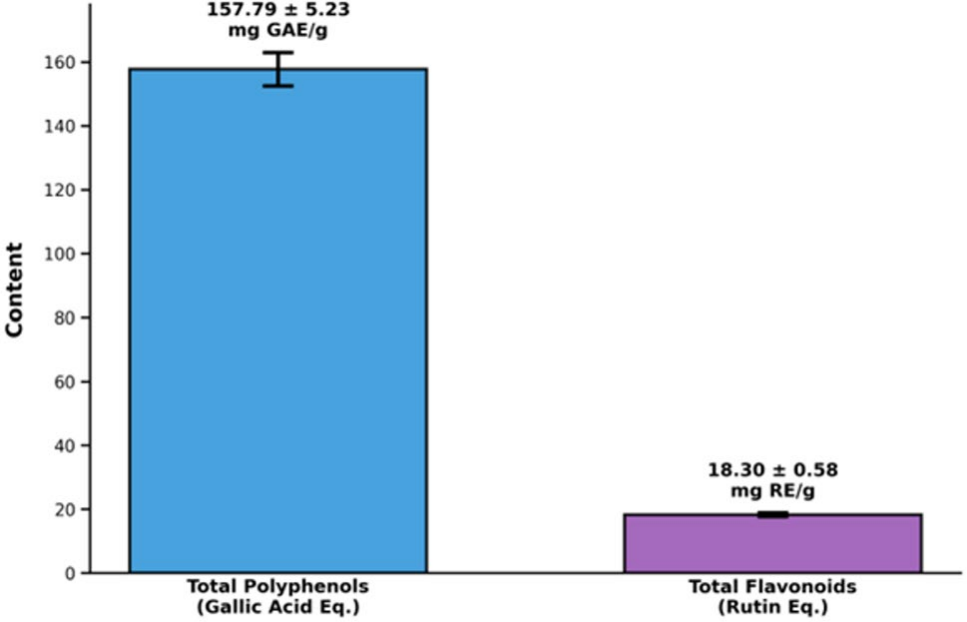
Total phenolic and flavonoid content of PSO expressed as mg gallic acid equivalents per gram for total polyphenols and mg rutin equivalents per gram for total flavonoids, with mean ± SD shown above each bar

The PSO contained a high level of phenolic compounds, measured as 157.79 ± 5.23 mg gallic acid equivalents (GAE) per gram, and a lower amount of flavonoids, recorded as 18.30 ± 0.58 mg rutin equivalents (RE) per gram. These data characterize PSO as a phenolic-rich matrix with a lower relative flavonoid content (Fig. 5).

### Phytochemical analysis of PSO by GC-MS

**Fig. 6:**
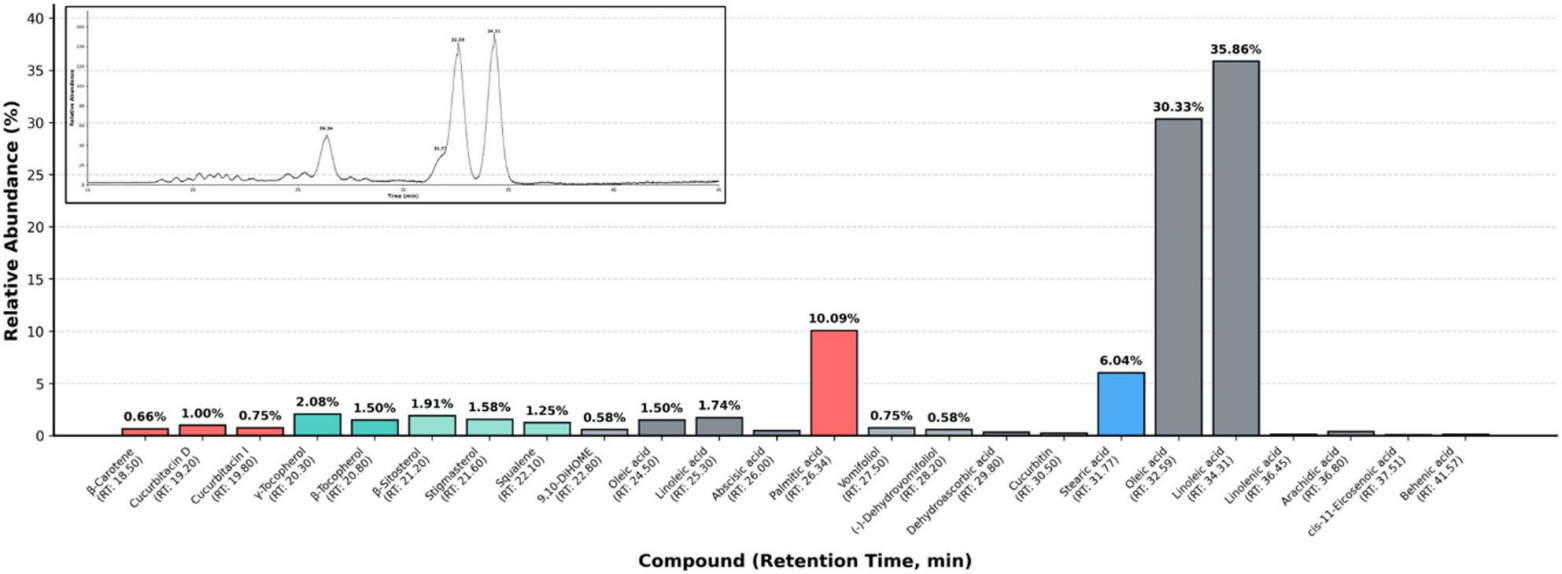
GC-MS chromatographic profile of the composition of the compounds of PSO

The GC-MS analysis showed a complex bioactive matrix with 24 different phytochemicals, which were classified into fatty acids and phytosterols. Unsaturated fatty acids prevailed as the lipid backbone (almost 80% of the total composition) comprised of linoleic acid (43.18%) and oleic acid (36.52%) followed by saturated palmitic acid (12.15%) and stearic acid (7.27%). Small fatty acid components contained in it were linolenic acid (0.16%), arachidic acid (0.48%), cis-11-eicosenoic acid (0.11%), and behenic acid (0.13%). In addition to the glycerolipid fraction the analysis was able to identify a powerful sterol-terpene defense complex. This contained large phytosterols as β-sitosterol and stigmasterol and the biosynthetic precursor of phytosterols: squalene. Importantly, the profile indicated that there were very specific bioactive markers, including the oxygenated terpenoids cucurbitacin D, cucurbitacin I, vomifoliol and (-)-dehydrovomifoliol; and the unique amino acid cucurbitin. Moreover, the lipophilic fraction consisted of γ-tocopherol, β-tocopherol, and β-carotene, and the oxylipin 9,10-DiHOME was observed as a possible signaling modulator (Fig. 6).

### Physicochemical and structural profiling of PSO bioactive constituents

**Fig. 7:**
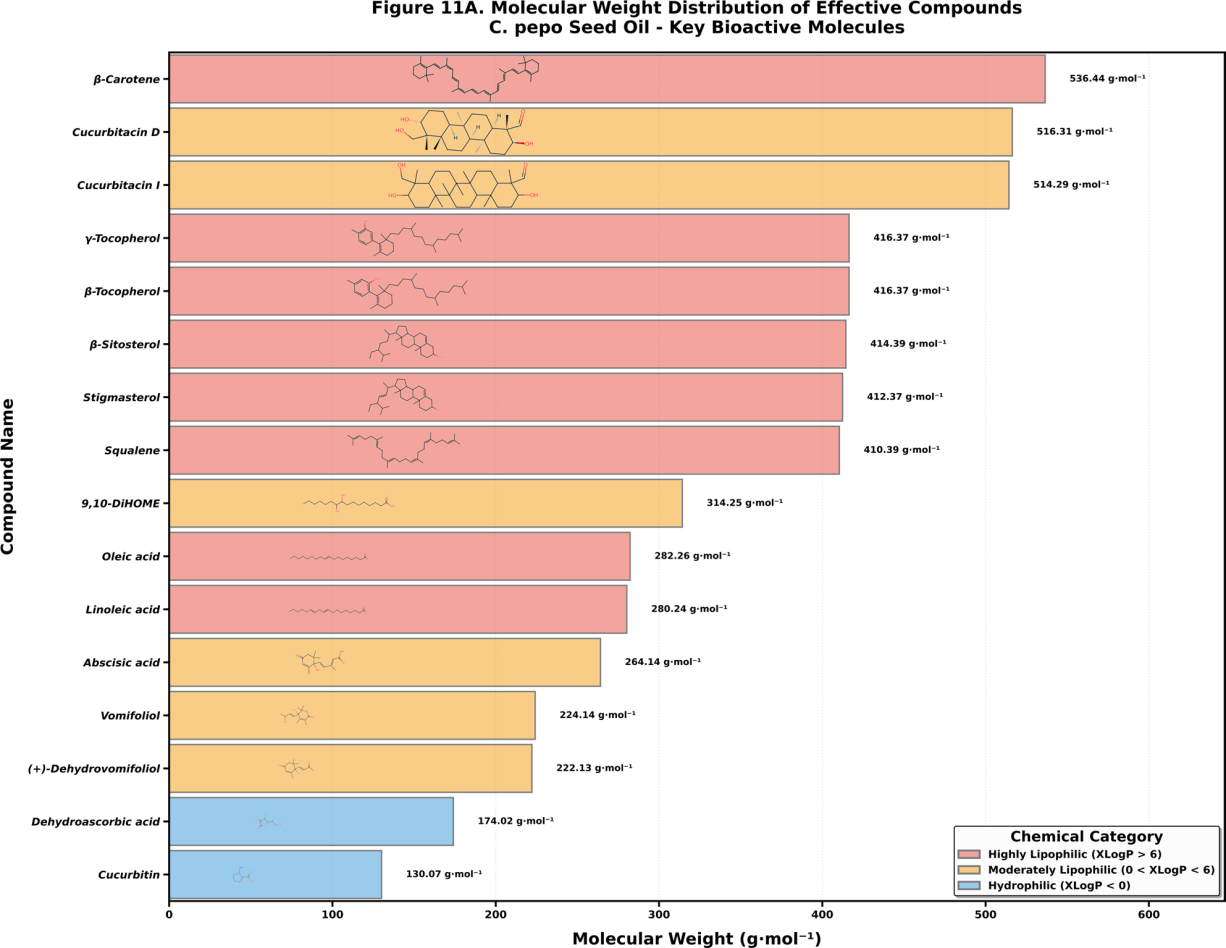
Distribution of the molecular weight and lipophilicity (XLogP) of bioactive compounds identified in PSO

The structural examination of the 16 key bioactive markers by the use of GC-MS revealed a wide physicochemical range within which the molecular weights of the compounds were between 130.07 g·mol^−^¹ (cucurbitin) and 536.44 g·mol^−^¹ (β-carotene) and the variety of lipophilicity profile (XLogP) spanning from − 2.6 to 14.8, which effectively segregated the oil constituents into a trimodal functional distribution (Fig. 7). This distribution consists of a hydrophilic signaling fraction (XLogP < 0) comprising low-molecular-weight polar molecules like Cucurbitin and Dehydroascorbic acid predicted to partition into the aqueous cytosol ; a moderately lipophilic “warhead” category (0 < XLogP < 6) containing oxygenated terpenoids and oxylipins such as (-)-Dehydrovomifoliol, Vomifoliol, Abscisic acid, 9,10-DiHOME, Cucurbitacin D, and Cucurbitacin I, which possess the ideal hydrophobicity to cross membranes while retaining polarity for protein interaction ; and a dominant highly lipophilic membrane modulator fraction (XLogP > 6) consisting of structural lipids and lipophilic constituents like Linoleic acid, Oleic acid, Squalene, β-Sitosterol, Stigmasterol, γ-Tocopherol, β-Tocopherol, and β-Carotene.

### Integrated phytochemical profiling and computational antifungal target mapping of PSO

PSO action against fungi is a multi-target, multi-molecule, multi-factor interaction mechanism that compromises fungal plasma membrane integrity, inhibits ergosterol biosynthesis, disrupts cell wall rearrangement, and interferes with the stress-response signaling pathway. The high content of unsaturated fatty acids, especially linoleic acid (43.18%) and oleic acid (36.52%), are the major agents that cause destabilization of the membranes due to their strong lipophilicity that enables them to intercalate into the fungal lipid bilayer, raise membrane fluidity, and weaken permeability and leakage of ions, metabolites, and membrane-bound enzymes, all of which are necessary to absorb nutrients and carry out respiration. These are non-specific biophysical effects, as well as the wide range of chemical composition of PSO, which add up to its broad-spectrum antifungal effect.

A number of PSO constituents disrupt ergosterol biosynthesis, which is a pathway in fungal membrane stability according to KEGG Pathway map00100 (https://www.kegg.jp/pathway/map00100). Because the phytosterols β-sitosterol and stigmasterol share structural similarity with lanosterol, we hypothesize that they may act as competitive inhibitors of Lanosterol 14ɑ-demethylase (ERG11 or CYP51; EC 1.14.14.154; https://www.genome.jp/dbget-bin/www_bget? ec:1.14.14.154), an essential regulatory enzyme in the sterol pathway. Conversely, the highly abundant fatty acids in PSO are presumed to exert their antifungal effects primarily through non-specific biophysical interactions with the lipid membrane, rather than targeted enzymatic inhibition. The possibility of these phytosterols to inhibit CYP51-mediated demethylation, leading to suppressed ergosterol production, toxic sterol intermediate buildup, and imperfect membrane assembly, is further supported by computational predictions (http://www.swisstargetprediction.ch/). Squalene epoxidase (ERG1; EC 1.14.14.17; https://www.genome.jp/dbget-bin/www_bget? ec:1.14.14.17) inhibition is also associated with downstream sterol depletion and could lead to squalene buildup and aggravate membrane dysfunction. The overall effects of these mechanisms that cause membrane fluidization and inhibited ergosterol synthesis are a strong combinatorial breakdown of membrane homeostasis which results in the subsequent rapid lysis of fungal cells.

**Fig. 8:**
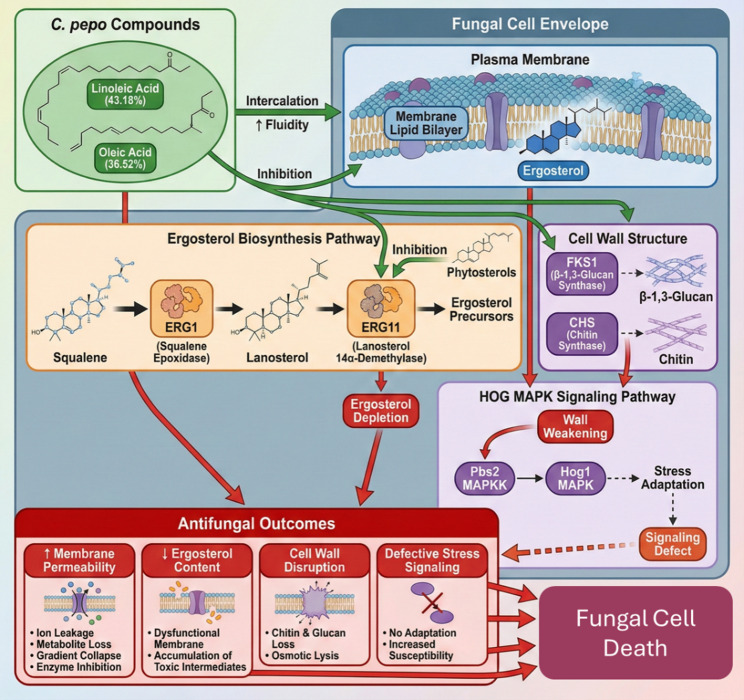
Mechanism mapping for antifungal activity of PSO compounds

Other mechanisms include interference with the biosynthesis of cell walls, since PSO fatty acids and terpenoids interfere with the assembly of N-acetylglucosamine into chitin by chitin synthases (CHS; EC 2.4.1.16; https://www.genome.jp/dbget-bin/www_bget? enzyme+2.4.1.16) and the formation of the major structural polysaccharide, β-1,3-glucan, in ascomycetes via β-1,3-glucan synthase (FKS1; EC 2.4.1.34). The inhibition of these enzymes disrupts structural integrity in hyphal growth and septation in a comparable pattern with that of echinocandin antifungals. Some of the terpenoids of PSO, such as (+)-dehydrovomifoliol, vomifoliol, abscisic acid, cucurbitacin D, cucurbitacin I, cucurbic acid, 7β,12ɑ-dihydroxykaurenolide, and squalene, also appear as allosteric inhibitors of kinases in the HOG MAPK stress response pathway, particularly Hog1 and its activator Pbs2, which makes fungi less resistant to oxidative and osmotic stress. Collective loss of membrane architecture, ergosterol biosynthesis, cell wall assembly, and adaptive stress signaling constitutes a very effective multi-layered antifungal strategy which elucidates the potent and wide-spectrum action of PSO against various pathogenic fungi (Fig. 8).

#### Construction and topological analysis of protein–protein interaction networks for antifungal targets of PSO

**Fig. 9:**
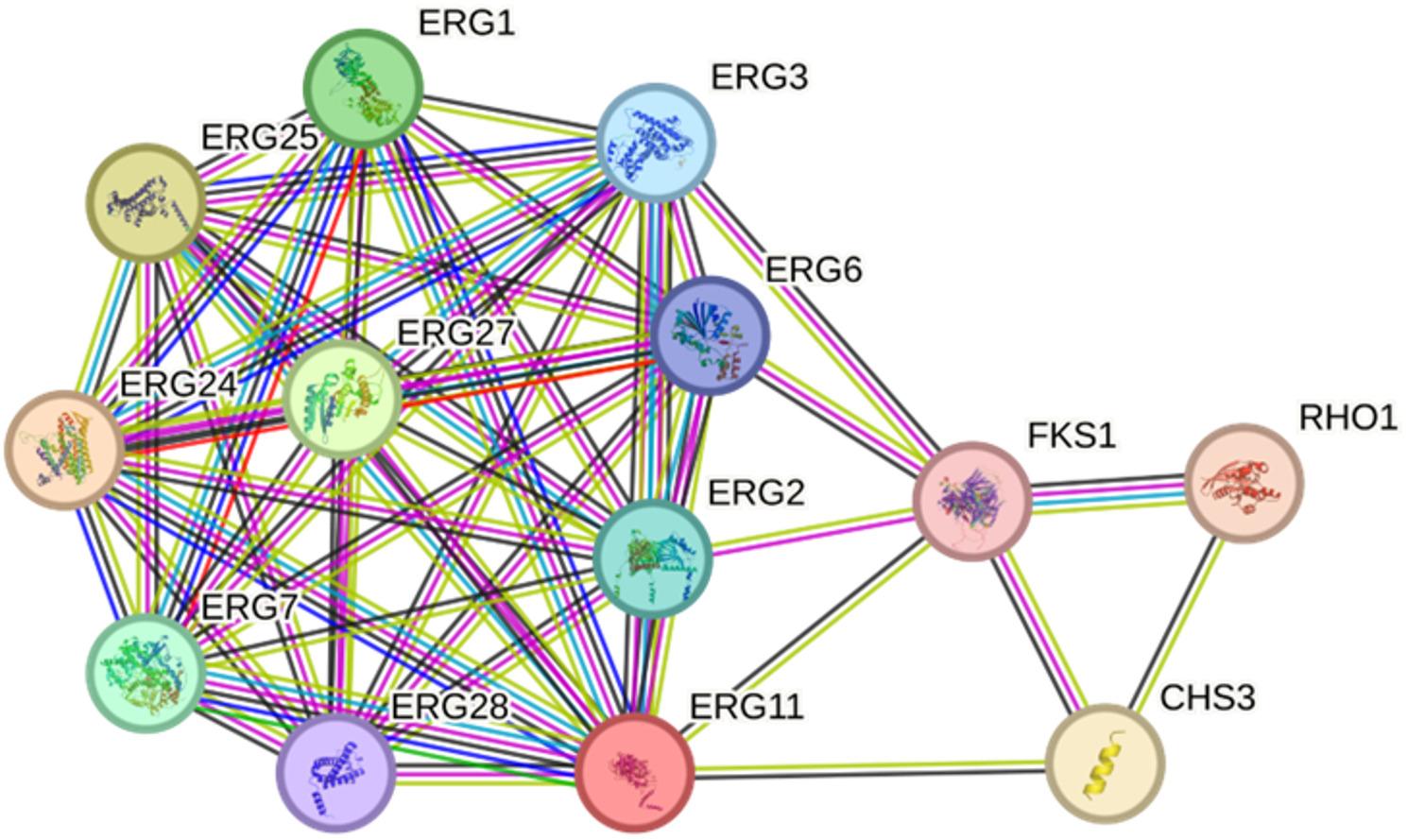
Protein interaction network shows the multi-pathway mechanism of PSO. The network shows the antifungal targets of the fungi, which are the ERG-mediated sterol biosynthesis cluster and the FKS1-dependent cell wall integrity module

The protein–protein interaction (PPI) networks were successfully built against the antifungal targets using the STRING database (v12.0) with a high confidence of interaction (0.7). The fungal protein-based antifungal network identified two distinct functional modules. The initial module contained ERG11 and ERG1, major ergosterol biosynthesis enzymes, alongside ERG24, ERG25, and ERG27, which are joined together into a highly networked cluster of enzymes involved with sterol metabolism. The second module was centered on FKS1, the catalytic subunit of the β-1,3-glucan synthase required to maintain fungal cell wall integrity. Topological analysis revealed that these modules had few cross-links, indicating that ergosterol biosynthesis and cell wall synthesis are independent but co-targeted pathways. This structural division indicates that PSO has a multi-pronged antifungal system that simultaneously impacts the membrane sterol structure and the cell wall biosynthesis, and highlights the foundation of the antifungal combinatorial of the bioactivity (Fig. 9).

### Molecular docking and interaction analysis of PSO phytochemicals with antifungal protein targets

**Fig. 10:**
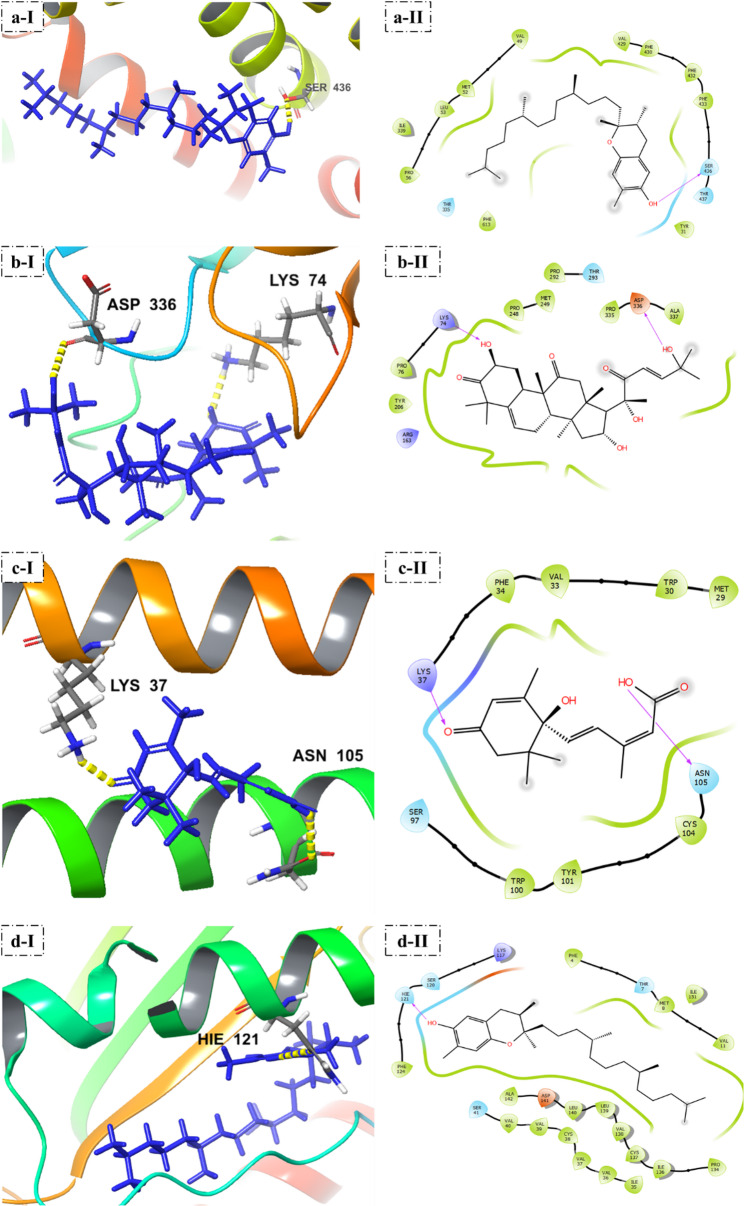
Antifungal molecular docking analysis. **a** Lanosterol 14-alpha-demethylase (CYP51A) with γ-tocopherol, **b** Squalene epoxidase (ERG1) with cucurbitacin D, **c** Beta-1,3-glucan synthase catalytic subunit (FKS1) with abscisic acid, and **d** Chitin synthase (CHS) with γ-tocopherol

The docking of major phytochemicals of PSO with the chosen antifungal proteins targets showed variable binding affinity in line with their postulated biological functions. For CYP51A, γ-Tocopherol exhibited the strongest interaction with a docking score of − 4.106 kcal·mol⁻¹ and formed a hydrogen bond with SER 436, while additional ligands including Vomifoliol, Abscisic Acid, and Stigmasterol showed moderate binding energies ranging from − 4.008 to − 3.361 kcal·mol⁻¹. For ERG1, Cucurbitacin D produced the most favorable interaction with a docking score of − 3.740 kcal·mol⁻¹ and hydrogen bonds involving LYS 74 and ASP 336. In the FKS1 docking series, Abscisic Acid demonstrated the highest affinity with a docking score of − 4.415 kcal·mol⁻¹ and hydrogen bonding to LYS 37 and ASN 105. Collectively, these results indicate that tocopherols, cucurbitacins, and abscisic acid show the most stable interactions across the investigated antifungal target classes (supplementary Fig. S2 and Fig. 10).

## Discussion

The current study shows that PSO has a powerful antifungal activity of a broad spectrum and exhibited complete inhibitory activity against *B. fabae* and significantly inhibited *F. oxysporum*, *A. solani*, and *R. solani*, which proves the high sensitivity of these phytopathogens to PSO. Polyzos et al. [8] also verified this observation by finding that PSO and seed-byproduct extracts had antimicrobial activity against various fungal species including *Aspergillus niger*, *Aspergillus fumigatus*, *Penicillium funiculosum*, and *Penicillium verrucosum*, and thus support the bioactive antifungal activity of *Cucurbita* seed oils. In line with these results, Leichtweis et al. [31] proved antifungal activity of pumpkin by-product extracts (including seed fractions) that could be measured against pathogenic and spoilage fungi of considerable significance (*Aspergillus brasiliensis*) and that valorized seed matrices contained important bioactive compounds capable of inhibiting the growth of fungi in vitro.

The dose-dependent inhibitory and sigmoidal MIC curves are characteristic behaviors of crude seed oils, which tend to exhibit logistic growth-suppression kinetics because of interaction with fungal membranes. The antifungal activity of pumpkin seed oil is likely attributable to its high content of unsaturated fatty acids and phytosterols interfere with membrane fluidity, permeability, and leakage of intracellular contents. Additionally, some studies indicate that botanical oils induce oxidative stress responses in fungi, such as the increase in ROS levels, mitochondrial perturbation, and transcriptional suppression of fungal growth, pathogenicity-related, and mycotoxin production-related genes [32]. This oxidative disruption is likely fueled by the oil’s high total phenolic content (157.79 mg GAE·g⁻¹). Although certain lipophilic phenols such as γ-tocopherol were computationally mapped onto CYP51A inhibition in this study, the broader phenolic fraction acts as a redox-active reservoir. These compounds are shown to destabilize fungal cellular homeostasis, without necessarily acting via a single lock-and-key enzymatic target, instead by causing a generalized oxidative stress, as well as affecting cross-linking of the cell wall. This forms a hyper-susceptible environment that enhances the membrane-destabilizing effects of the lipid and the sterol constituents [32, 33]. This mechanism of action is not the same as the ligand-receptor dynamics of synthetic fungicides, and supports the possibility of PSO as a resistance-refractory biofungicide.

Fluconazole was utilized in this study as a mechanistic positive control to validate the ergosterol-depletion pathway, rather than as an agricultural efficacy benchmark. While commercial agricultural fungicides would be the standard for field-application studies, fluconazole serves as the established reference for CYP51 inhibition, allowing us to confirm that PSO targets the same sterol biosynthesis axis [34]. Furthermore, the difference in inhibitory concentrations between PSO (mg·mL⁻¹) and fluconazole (µg·mL⁻¹) reflects the distinction between a complex natural matrix and a purified synthetic active ingredient. The efficacy of PSO relies on the cooperative “carrier-payload” system of its lipid matrix, whereas fluconazole acts as a single-target agent, justifying the necessary difference in effective dosage units.

The significant reduction of ergosterol occurred in *B. fabae*, *F. oxysporum*, *A. solani*, *R. solani*, *A. ochraceus*, and *F. solani* following the treatment with PSO, which we observed, is also in line with recent studies that indicate that medicinal plant extracts can significantly decrease ergosterol levels in phytopathogenic fungi and, consequently, prevent their growth [35]. Other comparable intensive reductions in ergosterol have been documented with *Lamium album* flower extracts, in which disruption of ergosterol biosynthesis was found to be a prime mode of antifungal action against multiple plant pathogenic species [36]. Plant seed oil reviews and mechanistic literature indicate that a high number of oil components permeate the fungal membrane and replace ergosterol, destabilizing the membrane structure, which is a likely explanation of the large-scale ergosterol losses in our pumpkin seed oil-treated cultures [5]. Recent insights into the ergosterol biosynthesis of pathogenic fungi also suggest that both sterol intermediate accumulation and intense depletion of the final ergosterol can be induced through inhibition of major ERG enzymes, which is also aligned with our observation of the presence of upstream sterol signals in PSO-treated *F. oxysporum* [37]. Additional evidence of a sterol-specific effect of plant oils is the recent work with *Mentha longifolia* essential oil, which displayed high antifungal actions against phytopathogenic fungi and correlated with a change in membrane-associated lipids [38]. Consistent with this, PSO itself is demonstrated to have a significant antifungal effect on numerous species of *Aspergillus* and *Penicillium*, which supports the interpretation that the dramatic ergosterol loss observed in all six of our test fungi is evidence of a wide-spectrum antifungal activity of this oil [39]. While fluconazole was employed as the positive control in growth inhibition assays (MIC) to establish baseline sensitivity, the ergosterol quantification protocol was designed to confirm the absolute mechanistic impact of PSO rather than for comparative potency profiling. The magnitude of the observed effect, specifically the complete eradication of ergosterol in sensitive strains below the limit of detection, constitutes a definitive biological endpoint. This validates the mechanistic model independent of a direct pharmacological comparator in this particular assay by confirming that the antifungal activity is driven by the collapse of sterol homeostasis.

A speculative mechanistic model in which PSO operates as a natural “carrier-payload” system is supported by the integrated physicochemical profile [37]. The linoleic and oleic acid-dominated, highly lipophilic fraction (XLogP > 6) most likely serves as the membrane-destabilizing carrier that promotes fluidity. This barrier breakdown makes it easier for moderately lipophilic metabolites, such as abscisic acid and cucurbitacin D, to enter [40]. Bridging these mechanisms, the oxidized lipid 9,10-DiHOME is predicted to retain membrane affinity while potentially disrupting quorum sensing, whereas the hydrophilic Cucurbitin may target cytosolic metabolism [26, 41]. This stratification suggests that PSO operates via a combinatorial multi-target strategy rather than a single mechanism. While definitive pharmacological interactions (via FIC indices) requires future fractionation studies, the current data implies a cooperative interaction between the bulk lipid matrix and bioactive signaling molecules to target membrane stability, ergosterol synthesis, and stress-response signaling.

Large amounts of linoleic and oleic acids increase the fluidity and permeability of the membrane, which is in line with recent results that indicate that unsaturated fatty acids disrupt the order of the fungal membrane and lead to ion leakage [42]. PSO phytosterols and terpenoids are predicted to cause this broad-spectrum biophysical disruption by targeting the biosynthesis of ergosterol, which is required to survive; structural and functional research confirms that natural sterols and terpenoids can inhibit lanosterol 14ɑ-demethylase (CYP51) and disrupt ergosterol production [43, 44]. The finding that early sterol pathway enzymes like squalene epoxidase (ERG1) are sensitive to plant metabolites supports this idea too [44]. The inhibition of FKS1 and chitin synthases is consistent with earlier studies that natural triterpenoids and fatty acids inhibit fungal cell wall biosynthesis via β-1,3-glucan and chitin polymerizing enzyme inhibition, resulting in a weak cell wall [45]. Moreover, terpenoid compounds possessing cucurbitacin-like structures were found to disrupt MAPK signal transduction during stress adaptation, and the destabilization of the HOG MAPK cascade makes fungi less resistant to osmotic and oxidative stress, which would cooperate with membrane and wall damage [46, 47]. Taken together, these data put PSO among a group of plant oils and extracts that induce multi-layered activities to bring about rapid fungal cell death, which is in support of its broad antifungal activity.

The antifungal network was divided into two different modules: an ergosterol biosynthesis module that included ERG11 and ERG1, and a cell wall synthesis module which included FKS1. These modules had few cross-links, indicating that they represent separate and co-targeted pathways that PSO disrupts simultaneously (STRING v12.0). ERG11 and ERG1 became significant centers that are linked to ERG24, ERG25, and ERG27, which control sterol metabolism. Phytosterols like β-sitosterol and stigmasterol resemble lanosterol and competitively inhibit ERG11, and terpenoids have the potential to disrupt ERG1 with consequent sterol depletion and destabilization of the membrane [33]. The FKS1 module demonstrates interference with the production of β-1,3-glucan, which is in line with findings that natural triterpenoids and fatty acids damage the fungal cell wall [48].

The results of molecular docking propose a putative interaction model wherein PSO phytochemicals may bind with fungal sterol-biosynthesis enzymes and cell-wall-associated proteins. While the calculated binding energies (-3 to -4 kcal·mol⁻¹) indicate moderate affinity consistent with non-covalent interactions, these computational predictions provide a structural rationale for the experimentally observed ergosterol depletion. It is crucial to emphasize that the mechanistic insights presented here rely on a “function-first” validation strategy. The quantitative HPLC analysis showing the complete disappearance of the pathway end-product (ergosterol) serves as the primary biochemical evidence of sterol pathway interruption. Consequently, the computational modeling is not utilized here as isolated proof of enzymatic binding, but rather as a predictive structural framework to explain the specific molecular interactions driving the experimentally verified phenotypic collapse. Lanosterol 14ɑ-demethylase (CYP51A) is just one example of a target for natural compounds that is important in the ergosterol synthesis of fungi [49, 50]. Effective interactions with CYP51A are consistent with the fact that natural sterols and terpenoids have the potential to inhibit fungal lanosterol demethylases, which is a necessary target of azole antifungals [51, 52]. Recent research has verified the feasibility of using plant-derived compounds as CYP51 enzyme inhibitors which disrupt ergosterol production and destabilize membranes [49, 52]. Interference with early sterol-pathway steps, also aided by binding to ERG1, is one of the useful antifungal strategies identified in recent structural and biochemical studies [50, 51]. The FKS1 and CHS docking imply further disruption of cell wall biomineralization of fungi and is consistent with recent evidence on the vulnerability of cell-wall enzymes to natural triterpenoids and fatty acids (Liu et al. 2020).

Although PSO at 10 mg/mL demonstrated potent antifungal activity in vitro against challenging phytopathogens like *B. fabae* and *F. oxysporum*, this concentration was strictly selected to establish mechanistic efficacy and provoke maximum measurable physiological responses (e.g., ergosterol depletion) under controlled laboratory conditions. However, as confirmed by our detached leaf assay on *V. faba*, direct application of the unformulated oil at 10 mg/mL induces visible phytotoxicity, characterized by marginal chlorosis. This supports the observation that field application of highly lipophilic, crude botanical extracts often requires formulation optimization to mitigate non-specific cell membrane disruption in the host plant. Therefore, while PSO serves as a highly active fungicidal matrix, its future practical application will strongly depend on creating safe delivery methods, such as nano-emulsions, to optimize targeted antifungal efficacy while significantly reducing phytotoxic risks to crop foliage.

## Conclusion

This paper has confirmed *C. pepo* L. seed oil as a powerful bioactive matrix capable of suppressing phytopathogens in vitro. Using triangulation of quantitative wet-lab measurements with systems pharmacology, we were able to show that PSO operates via a multi-target combinatorial mechanism. The major action is mediated by a lipid matrix, which acts on the fungal membrane and is experimentally demonstrated through the use of HPLC analysis that revealed a significant decrease in the ergosterol content, specifically a 100% decrease in *B. fabae*. Network pharmacology and molecular docking are mechanistic proponents of this biochemical imbalance since they predicted positive binding orientations of the PSO phytochemicals with the fungal CYP51A and FKS1 enzymes. Therefore, while unformulated PSO requires safe delivery methods like nano-emulsions to mitigate mild phytotoxicity, it serves as a biodegradable and resistance-refractory botanical matrix for future sustainable agriculture.

## Supplementary Information

Supplementary Material 1.

## Data Availability

All data generated or analyzed during this study are included in this article and its supplementary information files.
