# Supplementary material for: Antifungal efficacy of linoleic-acid-rich Cucurbita pepo L. seed oil revealed by GC-MS profiling of lipids and bioactive markers, ergosterol depletion, and network pharmacology
Source: BMC Microbiol. 2026 Mar 30;26:325. doi: 10.1186/s12866-026-04942-8 (PMC13063987; doi:10.1186/s12866-026-04942-8)
Supplement: Supplementary file 1 — Supplementary Material 1. [file 12866_2026_4942_MOESM1_ESM.docx]

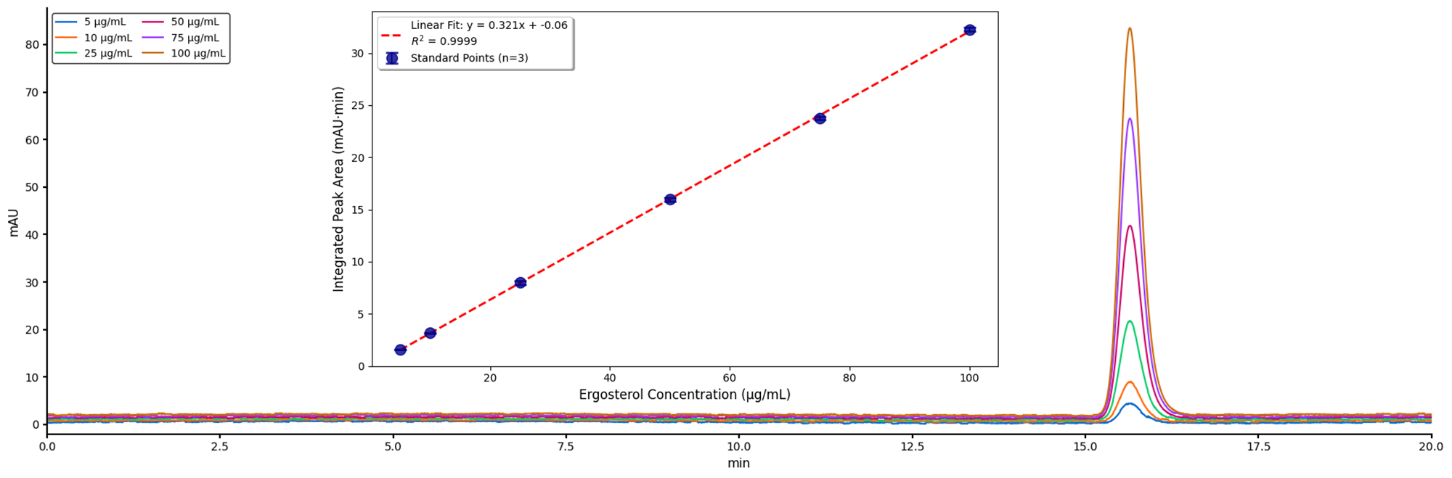


**Fig. S1** HPLC-DAD chromatograms of ergosterol standards and calibration curve

`
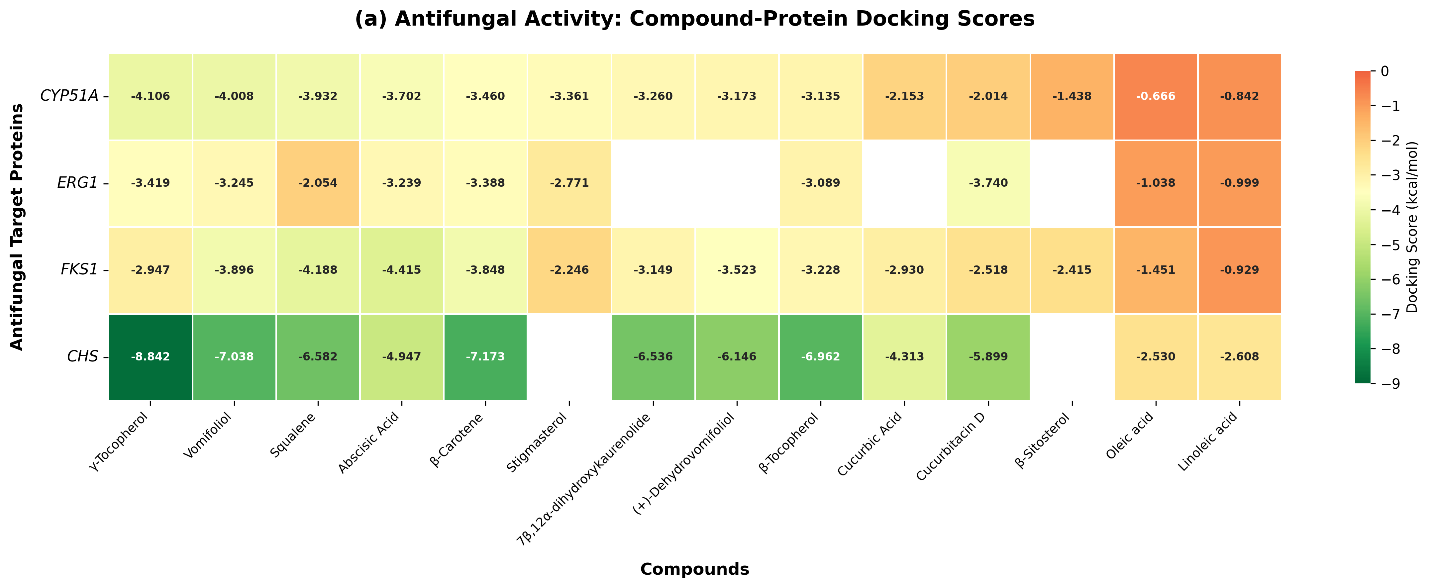


**Fig. S2** Heat map of docking scores of PSO compounds against antifungal target proteins
